# Radiotherapy for patients with locally advanced esophageal squamous cell carcinoma receiving neoadjuvant immunotherapy combined with chemotherapy

**DOI:** 10.1038/s41598-024-67419-6

**Published:** 2024-07-17

**Authors:** Yue Kong, Miaoyi Su, Jun Fang, Mengyuan Chen, Chao Zheng, Youhua Jiang, Kaiyi Tao, Changchun Wang, Guoqin Qiu, Yongling Ji, Yuezhen Wang, Yang Yang

**Affiliations:** 1 Department of Thoracic Radiation Oncology, Zhejiang Cancer Hospital, Hangzhou Institute of Medicine (HIM), Chinese Academy of Science, Hangzhou, 310022 China; 2 Department of Radiotherapy, Quanzhou Guangqian Hospital, Quanzhou, 362321 Fujian China; 3 Taizhou Campus of Zhejiang Cancer Hospital (Taizhou Cancer Hospital), Taizhou, 317502 Zhejiang China; 4 Department of Thoracic Surgery, Zhejiang Cancer Hospital, Hangzhou Institute of Medicine (HIM), Chinese Academy of Science, Hangzhou, 310022 China; 5 Wenling Institute of Big Data and Artificial Intelligence in Medicine, Taizhou, 317502 Zhejiang China

**Keywords:** Esophageal squamous cell carcinoma, Neoadjuvant chemoimmunotherapy, Radiotherapy, Treatment-related toxicity, Cancer, Cancer, Cancer therapy, Gastrointestinal cancer

## Abstract

With the success of immunotherapy in advanced esophageal cancer, neoadjuvant chemo-immunotherapy (CIT) is being increasingly used for local staged esophageal cancer, especially in the context of clinical trials, which brings similar pCR with neoadjuvant chemoradiotherapy and shows promising results. However, there is still a part of potentially operable patients can't undergo surgery after neoadjuvant chemo-immunotherapy. The follow-up treatment and prognosis of this population remain unclear. Patients pathologically diagnosed with ESCC, clinical stage T1-3N+M0 or T3-4aNanyM0 (AJCC 8th), PS 0–1 were retrospectively enrolled from 1/2020 to 6/2021 in Zhejiang Cancer Hospital. All patients firstly received PD-1 inhibitors plus chemotherapy (albumin paclitaxel, 260 mg/m^2^ on day 1 plus carboplatin AUC = 5 on day 1) every 3 weeks for 2–4 cycles. For those patients who did not receive surgery, definitive radiotherapy with 50.4 Gy/28F or 50 Gy/25F was adopted using VMAT, concurrent with chemotherapy or alone. The concurrent chemotherapy regimens included weekly TC (paclitaxel 50 mg/m^2^, d1, carboplatin AUC = 2, d1) or S1 (60 mg bid d1–14, 29–42). The survival outcomes and treatment toxicity were recorded and analyzed. A total of 56 eligible patients were finally identified from 558 patients who were treated in department of thoracic surgery, among all the patients, 25 (44.6%) received radiotherapy alone, and 31 (55.4%) received chemoradiotherapy after neoadjuvant CIT. The median follow-up was 20.4 months (interquartile range [IQR] 8.7–27 months). The median PFS and OS were 17.9 months (95% confidence interval [CI] 11.0–21.9 months) and 20.5 months (95% CI 11.8–27.9 months), respectively. In the subgroup analysis, the median OS was 26.3 months (95% CI 15.33–NA) for patients exhibiting partial response (PR) to CIT, compared to 17 months (95% CI 8.77–26.4) for those with stable disease (SD) or progressive disease (PD), yielding a hazard ratio (HR) of 0.54 (95% CI 0.27–1.06, P = 0.07). No significant difference was observed for patients received radiotherapy alone or chemoradiotherapy with HR = 0.73 (95% CI 0.72–2.6, P = 0.33). The most common Adverse events (AEs) observed during this study were anemia (98.2%), leukopenia (83.9%), thrombocytopenia (53.6%). AEs of grade ≥ 3 radiation-induced pneumonitis and esophagitis were 12.5% and 32.1%, especially, 6 patients (10.7%) died from esophageal fistula and 2 patients (3.6%) died from grade 5 pneumonitis. For local advanced ESCC patients after neoadjuvant CIT who did not receive surgery, definitive radiotherapy was an optional treatment strategy. However, those patients with no response to CIT also showed poor response to radiotherapy, and particular attention should be paid to treatment related toxicity, especially esophageal fistula.

## Introduction

Esophageal cancer represents a significant health burden in China, where it is the sixth most common gastrointestinal tumor in terms of incidence and ranks fourth in mortality among all malignant tumors^1^. Surgical intervention remains the cornerstone of treatment for patients with locally advanced esophageal cancer. Landmark clinical trials, such as the CROSS and NEOCRTEC 5010, have established that neoadjuvant chemoradiotherapy, when combined with surgery, enhances both overall survival (OS) and disease-free survival (DFS) compared to surgery alone. Despite these advancements, approximately 40% of patients still develop distant metastasis^2,3^. The JCOG 1109 trial highlighted that the DCF regimen (docetaxel, cisplatin, and 5-fluorouracil) surpasses neoadjuvant chemoradiotherapy in efficacy. However, it is associated with an increased risk of hematological toxicity^4^.

Immunotherapy has become a pivotal treatment modality across a spectrum of cancer types, demonstrating significant efficacy. Pioneering clinical trials, including JUPITER-06, KEYNOTE-590, CHECKMATE-648, ORIENT-15, and ESCORT-1, have reported objective response rates (ORR) ranging from 45 to 72% in esophageal cancer when employing chemoimmunotherapy as the first-line treatment^5–8^. Within the realm of neoadjuvant therapy, chemoimmunotherapy has shown to yield pathological complete response (pCR) rates comparable to those achieved with neoadjuvant chemoradiotherapy or chemotherapy alone^9–11^. Despite these advancements, a subset of patients who are potentially eligible for surgery encounter significant challenges in proceeding with surgical interventions after neoadjuvant chemoimmunotherapy. In fact, prospective clinical trial results show that approximately 20% of patients did not undergo surgical resection after neoadjuvant CIT^12–14^. For instance, only 36 of 45 (80.0%) patients underwent surgery after tislelizumab combined with chemotherapy in TD-NICE study^13^. The proportion of patients who do not receive surgery may be even higher in real-world practice. Most current studies focus on patients who successfully undergo surgery after neoadjuvant immunotherapy, while those who do not undergo surgery receive little attention. This underscores the necessity for continued research into alternative treatment strategies and the prognosis for this distinct patient cohort.

Considering these factors, our study aimed to evaluate patients with locally advanced esophageal squamous cell carcinoma who were deemed unresectable after receiving neoadjuvant chemoimmunotherapy. Our objective was to identify the most effective treatment strategy for these individuals, with a specific focus on assessing the safety and efficacy of alternative therapeutic approaches.

## Methods

### Patients

Patients with pathologically confirmed esophageal squamous cell carcinoma (ESCC) at clinical stages T1-3N+M0 or T3-4aNanyM0 (according to the AJCC 8th edition) and an Eastern Cooperative Oncology Group Performance Status (ECOG PS) of 0–1 were consecutively included in a retrospective study conducted at Zhejiang Cancer Hospital from January 2020 to June 2022. Initially, all patients underwent treatment with PD-1 inhibitors (Camrelizumab, Sintilimab, or Tislelizumab) in combination with chemotherapy (albumin paclitaxel, 260 mg/m^2^ on day 1, and carboplatin AUC = 5 on day 1) administered every 3 weeks for 2–4 cycles. The inclusion criteria of the study were patients that were still ineligible for surgery due to various reasons, after receiving neoadjuvant immunotherapy combined with chemotherapy.

For those patients, alternative definitive radiotherapy was administered at a dose of 50.4 Gy/28F or 50 Gy/25F using Volumetric Modulated Arc Therapy (VMAT) or Intensity-Modulated Radiation Therapy (IMRT), either concurrently with chemotherapy or alone. Concurrent chemotherapy regimens consisted of weekly TC (paclitaxel 50 mg/m^2^ on day 1 and carboplatin AUC = 2 on day 1) or S1 (60 mg twice daily on days 1–14 and 29–42). Survival outcomes and treatment-related toxicities were comprehensively documented and analyzed.

### Data collection and endpoints

The study's dual primary endpoints encompass overall survival (OS) and progression-free survival (PFS). OS is defined as the duration from treatment initiation to the occurrence of death or last follow-up, while PFS is delineated as the period from treatment commencement to the initial manifestation of local failure, metastatic recurrence, disease progression, or mortality. Evaluation of primary tumor response is conducted via CT scan utilizing the Response Evaluation Criteria in Solid Tumors (RECIST) version 1.1, with outcomes classified as Complete Response (CR), Partial Response (PR), Stable Disease (SD), or Progression Disease (PD). The objective response rate (ORR) denotes the proportion of patients achieving CR or PR, whereas the disease control rate (DCR) signifies the ratio of patients achieving CR, PR, or SD. The secondary endpoint involves documenting adverse events associated with chemoradiotherapy, including radiation esophagitis, radiation pneumonitis, and hematologic toxicity, graded in accordance with the Radiation Therapy Oncology Group Criteria for Adverse Events.

### Statistical analysis

Overall survival, progression-free survival, and duration of response were estimated using the Kaplan–Meier method. All reported p-values were two-sided, with statistical significance set at p < 0.05. Statistical analysis was performed using R software (version 4.1.2; R Foundation for Statistical Computing, Vienna, Austria; https://www.R-project.org/).

### Ethical statement

This study adhered to the principles outlined in the Declaration of Helsinki and met all relevant requirements in China. Approval for the study was obtained from the Zhejiang Cancer Hospital Ethics Committee (approval number: IRB-2021-94). Given the retrospective nature of the study, the need for informed consent was waived by the institutional review boards.

## Results

### Patient characteristics

**Figure 1:**
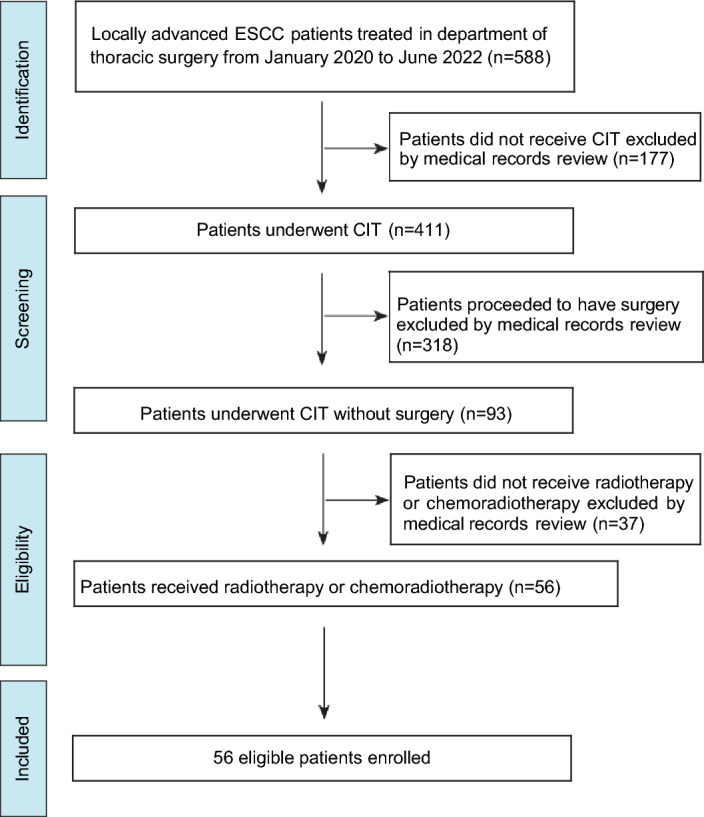
Flowchart of patient selection.

Out of 558 patients treated in the Department of Thoracic Surgery, 56 met the eligibility criteria for this study. Among them, 31 patients exhibited no response to neoadjuvant chemoimmunotherapy (CIT), with 6 demonstrating progression of disease (PD) and 25 showing stable disease (SD). Additionally, 25 patients achieved a partial response (PR) but did not undergo surgery due to either poor performance status or refusal to undergo the operation (see Fig. 1). Initially, 7 patients had diabetes, 9 had hypertension, and 4 had interstitial pneumonia. 11 patients had an NRS2002 score of ≥ 3 at the initial treatment stage, 18 patients had an NRS2002 score of ≥ 3 after nCIT.

The median age of the cohort was 66 years (interquartile range [IQR] 56–72), with 55 individuals (98.2%) being male. Regarding disease staging, 12 patients (19.6%) were classified as stage II, while 44 patients (80.4%) were classified as stage III before treatment. After neoadjuvant CIT, 4 patients experienced primary lesion progression (T4), and 2 patients had lymph node progression. Among all patients, 25 (44.6%) received radiotherapy alone, while 31 (55.4%) received chemoradiotherapy following neoadjuvant chemoimmunotherapy (refer to Table 1). After chemoradiotherapy or radiotherapy, four patients were re-evaluated as PR and recommended for surgery. However, due to poor physical condition or refusal of surgery, none of these patients underwent the procedure. Instead, we recommended a strategy of close monitoring and regular follow-ups for these patients.

**Table 1:** Patient characteristics.

Factors	No. (%) of all patients (N = 56)
Age	
Median (IQR) years	66 (56–72)
Sex	
Female	1 (1.8)
Male	55(98.2)
ECOG	
1	54 (96.4)
2	2 (3.6)
Smoking	
No	13 (23.2)
Yes	43 (76.8)
Drinking	
No	13 (23.2)
Yes	43 (76.8)
NRS-2002 score	
≥ 3	11 (19.6)
< 3	45 (80.4)
Comorbidities	
Diabetes	4 (7.1%)
Hypertension	6 (10.7%)
Chronic bronchitis/emphysema	4 (7.1%)
Heart disease	1 (1.8%)
Clinical T stage	
T1	1 (1.8%)
T2	16 (28.6%)
T3	29 (51.8%)
Clinical N stage	
N0	1 (1.8%)
N1	23 (28.6%)
N2	22 (39.3%)
Clinical stage	
II	12 (19.6)
III	44 (80.4)
Location	
Upper	15 (26.8)
Mid	21 (37.5)
Lower	20 (35.7)
Overall response to IO + chemo	
PR	25 (44.6)
SD	25 (44.6)
PD	6 (10.7)
Treatment patterns	
RT alone	25 (44.6)
CRT	31 (55.4)

*IQR* interquartile range, *ECOG* Eastern Cooperative Oncology Group, *PR* partial response, *SD* stable disease, *PD* progression disease, *RT* radiotherapy, *CRT* chemoradiotherapy.

### Survival

**Figure 2:**
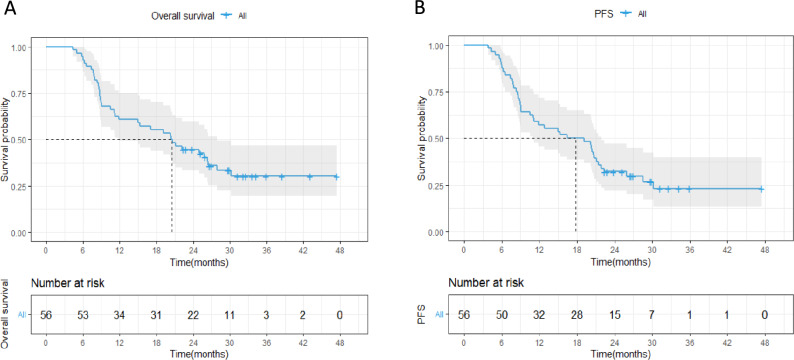
Overall survival and progression free survival outcomes for the entire cohort.

As depicted in Fig. 2, the median follow-up duration was 20.4 months (interquartile range [IQR] 8.7–27 months). Median progression-free survival (PFS) and overall survival (OS) for the cohort stood at 17.9 months (95% confidence interval [CI] 11.0–21.9 months) and 20.5 months (95% CI 11.8–27.9 months), respectively.

**Figure 3:**
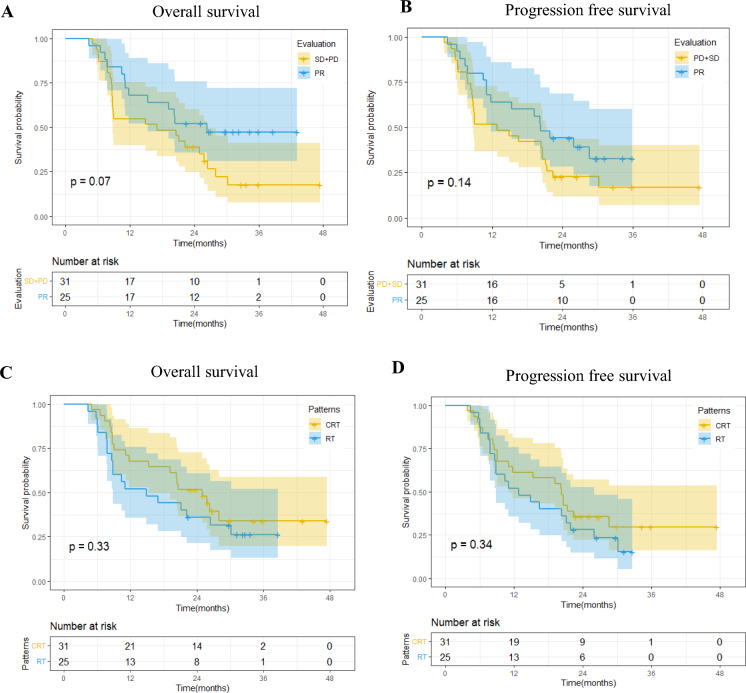
Subgroup analyses by therapeutic efficacy of neoadjuvant chemoimmunotherapy and subsequent treatment modalities.

Subgroup analysis revealed a median PFS of 20.3 months (95% CI 11.83-NA) for patients exhibiting partial response (PR) to CIT, compared to 12.8 months (95% CI 8.67–21.4) for those with stable disease (SD) or progressive disease (PD), yielding a hazard ratio (HR) of 0.62 (95% CI 0.33–1.17, P = 0.14). Median OS for patients with PR to CIT was 26.3 months (95% CI 15.33–NA), contrasting with 17 months (95% CI 8.77–26.4) for SD or PD patients, with an HR of 0.54 (95% CI: 0.27–1.06, P = 0.07) (as shown in Fig. 3A,B).

For patients undergoing radiotherapy alone compared to those receiving chemoradiotherapy, the median PFS was 12.8 months (95% CI 8.83–25.9) and 20.3 months (95% CI 11.03–NA), respectively, resulting in an HR of 0.74 (95% CI 0.73–2.5, P = 0.34). Similarly, the median OS for radiotherapy alone was 15 months (95% CI 8.83–NA), while for chemoradiotherapy, it was 25 months (95% CI 15.33–NA), with an HR of 0.73 (95% CI 0.72–2.6, P = 0.33) (As shown in Fig. 3C,D).

### Patterns of failure

**Figure 4:**
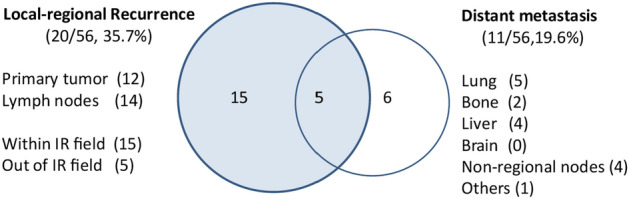
Recurrence patterns.

A total of 26 patients encountered recurrence during the study period. Specifically, 20 patients manifested local recurrence, 12 exhibited recurrence of the primary esophageal lesion, while 14 presented with lymph node recurrence. Additionally, 15 cases demonstrated recurrence within the irradiated field (IR), whereas 5 patients experienced recurrence outside the original IR field. Distant metastasis was documented in 11 patients, predominantly affecting sites such as the lung, liver, distant lymph nodes, bone, and other organs, with varying degrees of incidence, as illustrated in Fig. 4. In the RT group, 11 out of 25 patients experienced recurrence or metastasis. Among these, 9 patients had local regional recurrences, 3 patients had distant metastases, and 2 patients had both recurrence and metastasis. In the CRT group, 15 out of 31 patients experienced recurrence or metastasis (P = 0.73, compared to the RT group). Among these, 11 patients had local regional recurrences (P = 0.96, compared to the RT group), and 9 patients had distant metastases (P = 0.12, compared to the RT group), involving organs such as the lungs, liver, and bones. Additionally, 5 patients had both recurrence and metastasis.

### Toxicity effects

During neoadjuvant CIT, the main adverse events (AEs) were Grade 1–2, additionally, two patients experienced Grade 3 heart injury, and one patient developed Grade 3 immune-related enterocolitis. During the course of chemoradiotherapy or radiotherapy, prevalent adverse eventsincluded anemia (98.2%), leukopenia (83.9%), and thrombocytopenia (53.6%). Furthermore, grade ≥ 3 radiation-induced pneumonitis and esophagitis were noted in 12.5% and 32.1% of cases, respectively. Notably, complications such as esophageal fistula led to fatalities in 6 patients (10.7%), while 2 patients (3.6%) succumbed to grade 5 pneumonitis (Table 2). Consequently, heightened vigilance is warranted in subsequent treatment protocols to mitigate such risks.

**Table 2:** Adverse events during neoadjuvant chemotherapy and immunotherapy and definitive chemoradiotherapy.

Grade of adverse event, no. (%)	Grade 1	Grade 2	Grade 3	Grade 4	Grade 5
Immune-related adverse events during nCIT					
Rash	1 (1.7)	0	0	0	0
Thyroid function abnormalities	5 (8.9)	0	0	0	0
ALT/AST abnormalities	10 (17.9)	0	0	0	0
Renal function abnormalities	4 (7.1)	0	0	0	0
Cardiac arrhythmia	6 (10.7)	0	0	0	0
Fatigue	10 (17.9)	0	0	0	0
Decreased appetite	14 (25)	0	0	0	0
Myocarditis	0	0	2 (3.6)	0	0
Enterocolitis	0	0	1 (1.7)	0	0
Pneumonitis	0	1 (1.7)	0	0	0
Adverse events during definitive chemoradiotherapy/radiotherapy					
Leukopenia	12 (20.7)	24 (41.4)	11 (19.0)	0	0
Thrombocytopenia	7 (12.1)	16 (37.6)	6 (10.3)	1 (1.7)	
Anemia	25 (43.1)	22 (37.9)	7 (12.1)	1 (1.7)	
Radiation esophagitis	21 (37.5)	16 (28.6)	8 (14.3)	4 (7.2)	6 (10.7)
Radiation pneumonitis	14 (25.0)	14 (25.0)	3 (5.4)	2 (3.6)	2 (3.6)

## Discussion

Esophageal cancer ranks among the prevalent gastrointestinal tumors in China. Surgical intervention represents a cornerstone in the management of locally advanced esophageal cancer (ESCC), boasting a 5-year overall survival rate of 52.9%. However, post-surgery recurrence remains a significant challenge, affecting approximately 33.7% of patients^1^. Even with the consideration of neoadjuvant therapy for potentially operable patients falling under the cT1-3N+M0 or cT3-4aNanyM0 categories according to the 8th UICC/AJCC staging system, treatment outcomes often fall short of expectations due to relapse or hematologic toxicity^2^. Consequently, there arises a critical need to explore novel neoadjuvant strategies characterized by enhanced efficacy and diminished side effects, representing the next frontier in ESCC management.

Immunotherapy has emerged as a transformative approach in the treatment landscape of various malignancies, encompassing melanoma, lung cancer, kidney cancer, and lymphomas^15–18^. Notably, the integration of immune checkpoint inhibitors (ICIs) with chemotherapy has yielded enhanced clinical outcomes across diverse squamous cell malignancies^19–21^. Metastatic esophageal squamous cell carcinoma has also witnessed substantial clinical benefits from ICIs, as evidenced by pivotal trials such as JUPITER-06, KEYNOTE-590, CHECKMATE-648, ORIENT-15, and ESCORT-1^22^. Concurrently, several phase II clinical trials have investigated the neoadjuvant combination of PD-L1 inhibitors and chemotherapy for locally advanced squamous esophageal cancer, collectively underscoring its efficacy and tolerability in this setting^9–11^. Despite limitations in patient populations, the treatment outcomes have been promising. Nevertheless, a subset of patients may remain unresectable following neoadjuvant therapy, with the proportion potentially higher in real-world scenarios. Such individuals may subsequently undergo chemoradiotherapy or radiotherapy. The landmark RTOG-8501 trial demonstrated that combined therapy improved survival outcomes among patients with T1-3N0-1M0 esophageal cancer, with a 5-year overall survival ranging from 14 to 26% in the concurrent chemoradiotherapy group^23^. However, it's important to note that severe acute toxic effects were more pronounced in the combined therapy cohort, with only 68% of patients able to complete chemotherapy as planned, and 10% experiencing life-threatening toxic effects. Moving forward, it is imperative to assess radiotherapy as an alternative treatment modality for patients who remain ineligible for surgery following neoadjuvant immunotherapy combined with chemotherapy.

In our study, we initially enrolled 588 patients, among whom 411 underwent neoadjuvant chemoimmunotherapy, while 318 proceeded to surgery. The remaining 56 patients received either radiotherapy alone or chemoradiotherapy. The 2-year overall survival (OS) rate was notably lower at 37.8% for this subset compared to patients receiving concurrent treatment modalities. This suggests the existence of a specific subgroup of esophageal cancer patients who may exhibit insensitivity to chemotherapy, immunotherapy, and radiotherapy. Indeed, emerging research has shed light on the intricate interplay between the immune status within the tumor microenvironment (TEM) and the efficacy of chemotherapy or radiation therapy^24,25^. Factors such as hypoxia, tumor stroma, and metabolic reprogramming have been identified as key contributors to this dynamic, with ongoing studies elucidating the underlying mechanisms and identifying potential therapeutic targets for clinical intervention^26–29^. However, it is essential to recognize that these patients often face a poorer prognosis and heightened risk of severe side effects. Sequential administration of immunotherapy and radiotherapy may exacerbate the risk of esophageal fistula, as evidenced by our observed fistula rate of 10.7%. This underscores the importance of meticulous delineation of target volumes and judicious selection of chemotherapy regimens to mitigate treatment-related toxicity. In subgroup analysis, although not statistically significant, a discernible trend towards improved outcomes was observed in the partial response (PR) group receiving chemoimmunotherapy compared to those with progressive disease (PD) or stable disease (SD). Additionally, chemoimmunotherapy effectively curtailed recurrence within the local region. Conversely, no discernible difference was noted between patients receiving radiotherapy alone and those undergoing chemoradiotherapy, indicating a potential insensitivity to radiotherapy in this patient cohort.

We must acknowledge several limitations inherent in our study. Firstly, it was a single-center retrospective study, susceptible to potential selection bias and recall bias. For instance, some patients received radiotherapy as outpatients and did not continue their care at our hospital. Consequently, the information provided by their family members during follow-up calls might be inaccurate. Secondly, the sample size was relatively small, thereby limiting the feasibility of conducting further subgroup analyses and resulting in insufficient statistical power to draw definitive conclusions. In addition, some clinical or laboratory factors were not included in the analysis, resulting in unclear confounding effects. Lastly, the follow-up period was relatively short, with overall survival (OS) not yet reached for parts of the patients. Also, variations in the patients' subsequent systemic treatments influenced the overall survival outcomes in the conclusions. However, despite these limitations, our real-world study provides valuable and up-to-date information, including prognosis and safety data, regarding definitive radiotherapy for patients following combined immunotherapy and chemotherapy. Additionally, our findings underscore the importance for radiation oncologists to remain vigilant for severe esophageal fistula and pneumonitis in clinical practice.

Our study found no difference in prognosis between the RT and CRT groups for this cohort. Clinical oncologists should approach this conclusion with caution, particularly when applying it in a clinical setting. Multiple factors may have influenced our findings. Firstly, the prior neoadjuvant chemotherapy combined with immunotherapy might have diminished the impact of concurrent chemotherapy. Secondly, the fact that this group of patients could not undergo surgery even after receiving neoadjuvant chemotherapy combined with immunotherapy suggests that they were not responsive to systemic treatment and may also have developed resistance to immunotherapy. Additionally, their poor performance scores likely weakened the benefits of CRT and increased the side effects. Thirdly, variations in the patients' subsequent systemic treatments after CRT/RT may have influenced the overall survival outcomes, contributing to potential inaccuracies in our conclusions. Lastly and most importantly, the small sample size likely contributed to the absence of a statistically significant difference between the CRT and RT groups, even though the survival rate of CRT patients was better than that of RT patients. A future prospective trial with a larger sample size is needed to clarify this issue.

In conclusion, our study presents, for the first time, survival outcomes and treatment-related adverse effects in patients with esophageal squamous cell carcinoma (ESCC) who underwent radiotherapy following neoadjuvant CIT, with no prior related reports available. Overall, for locally advanced ESCC patients who did not undergo surgery after neoadjuvant CIT, definitive radiotherapy emerged as a viable treatment option. However, individuals showing poor response to CIT also exhibited limited efficacy with radiotherapy, and careful monitoring of treatment-related toxicity is needed, especially for esophageal fistula. Prospective, ideally randomized clinical trials are warranted to identify the most efficacious treatment modalities for such patients and validate our findings.

## Data Availability

All data and material are available from the corresponding author on reasonable request. The datasets supporting the conclusions of this article are included within the article.

## Abbreviations

CIT: Chemoimmunotherapy
CR: Complete response
CRT: Chemoradiotherapy
DCF: Docetaxel + cisplatin + 5-fluorouracil
DCR: Disease control rate
DFS: Disease-free survival
ECOG: Eastern cooperative oncology group
ESCC: Esophageal squamous cell carcinoma
IQR: Interquartile range
ORR: Objective response rate
OS: Overall survival
pCR: Pathological complete response
PD: Progression disease
PFS: Progression free survival
PR: Partial response
SD: Stable disease
